# Automated peritoneal lavage: an extremely rapid and safe way to induce hypothermia in post-resuscitation patients

**DOI:** 10.1186/cc12518

**Published:** 2013-02-20

**Authors:** Monique C de Waard, Hagen Biermann, Stijn L Brinckman, Yolande E Appelman, Ronald H Driessen, Kees H Polderman, Armand RJ Girbes, Albertus Beishuizen

**Affiliations:** 1 Department of Intensive Care, Institute for Cardiovascular Research, VU University Medical Center, De Boelelaan 1117, Amsterdam, 1007 MB, The Netherlands; 2 Department of Cardiology, Institute for Cardiovascular Research, VU University Medical Center, De Boelelaan 1117, Amsterdam, 1007 MB, The Netherlands; 3 Department of Critical Care Medicine, University of Pittsburgh Medical Center, 3550 Terrace Street, 601A Scaife Hall, Pittsburgh, PA, USA

## Abstract

**Introduction:**

Mild therapeutic hypothermia (MTH) is a worldwide used therapy to improve neurological outcome in patients successfully resuscitated after cardiac arrest (CA). Preclinical data suggest that timing and speed of induction are related to reduction of secondary brain damage and improved outcome.

**Methods:**

Aiming at a rapid induction and stable maintenance phase, MTH induced via continuous peritoneal lavage (PL) using the Velomedix^® ^Inc. automated PL system was evaluated and compared to historical controls in which hypothermia was achieved using cooled saline intravenous infusions and cooled blankets.

**Results:**

In 16 PL patients, time to reach the core target temperature of 32.5°C was 30 minutes (interquartile range (IQR): 19 to 60), which was significantly faster compare to 150 minutes (IQR: 112 to 240) in controls. The median rate of cooling during the induction phase in the PL group of 4.1°C/h (IQR: 2.2 to 8.2) was significantly faster compared to 0.9°C/h (IQR: 0.5 to 1.3) in controls. During the 24-hour maintenance phase mean core temperature in the PL patients was 32.38 ± 0.18°C (range: 32.03 to 32.69°C) and in control patients 32.46 ± 0.48°C (range: 31.20 to 33.63°C), indicating more steady temperature control in the PL group compared to controls. Furthermore, the coefficient of variation (VC) for temperature during the maintenance phase was lower in the PL group (VC: 0.5%) compared to the control group (VC: 1.5%). In contrast to 23% of the control patients, none of the PL patients showed an overshoot of hypothermia below 31°C during the maintenance phase. Survival and neurological outcome was not different between the two groups. Neither shivering nor complications related to insertion or use of the PL method were observed.

**Conclusions:**

Using PL in post-CA patients results in a rapidly reached target temperature and a very precise maintenance, unprecedented in clinical studies evaluating MTH techniques. This opens the way to investigate the effects on neurological outcome and survival of ultra-rapid cooling compared to standard cooling in controlled trials in various patient groups.

**Trial Registration:**

ClinicalTrials.gov: NCT01016236

See related letter by Esnault *et al., *http://ccforum.com/content/17/3/431

## Introduction

Most patients who suffer a cardiac arrest (CA) do not survive, and full neurological recovery occurs in only 6 to 23% [1]. Mild therapeutic hypothermia (MTH) is nowadays an established treatment to limit neurological injury and to improve outcome in CA patients after successful resuscitation [2,3]. Based on two prospective clinical trials [2,3], the International Liaison Committee on Resuscitation has recommended that all unconscious adult patients with spontaneous circulation after out-of-hospital CA as a result of ventricular fibrillation should be cooled to 32 to 34°C for 12 to 24 hours [4]. Many techniques for inducing and maintaining hypothermia and for controlled re-warming have been described [5-7]. However, the rate of lowering body temperature to achieve target temperature and the stability of target temperature during the maintenance phase still need to be optimized [3,4,8,9]. The clinical benefits of intervening in the process of ischemic and reperfusion injury using MTH are thought to be greatest when achieved as early and as fast as possible. Although pre-clinical animal studies have shown beneficial effects of starting prior to or during early reperfusion with therapeutic hypothermia [10-12], translation of the approach of early cooling to humans is still difficult. Interestingly, Bernard *et al. *[8] found that a fast cooling rate of 1.6°C/h using rapid intravenous infusion of cold lactated Ringers solution, in 22 comatose patients following resuscitation from CA, was safe and effective. Compared to historical controls, outcome was significantly improved with no increase in complications. Howes *et al. *[13] achieved rapid induction of therapeutic hypothermia with a cooling rate of 3.0°C/h using convective-immersion surface cooling resulting in an excellent survival rate. In contrast, a faster decline in body temperature to 34°C appears to predict an unfavourable neurological outcome in a study by Haugk *et al. *[14]. Although (pre-) clinical studies strongly suggest that timing and speed of induction of MTH are related to reduction of secondary brain damage and improved outcome, translation to improved clinical care has not yet been established.

As part of a multi-center trial, we tested a new application of MTH rapidly induced via peritoneal lavage. The equipment used is a modification of existing technologies and standard techniques which are used on a daily basis in peritoneal dialysis and laparoscopic surgery [15]. The system is designed to induce, maintain and reverse MTH by continuous lavage of the peritoneal cavity with lactated Ringers solution. This article reports preliminary results on the safety and feasibility of inducing MTH using peritoneal lavage compared with conventional cold, intravenous infusion and cooling blankets.

## Materials and methods

### Patients in the peritoneal lavage group

As part of a multi-center prospective observational study, we tested a new application to induce MTH using the Velomedix^® ^Inc. automated peritoneal lavage (PL) system (San Francisco, CA, USA). The Velomedix automated PL system is an investigational device from the United States, limited by Federal (United States) law to investigational use. In our institute (VU University Medical Center, Amsterdam, The Netherlands), 16 unconscious, mechanically ventilated patients with return of spontaneous circulation (ROSC) after CA requiring standard treatment with MTH were admitted to the ICU between November 2010 and July 2011, and included in the PL group. The research ethics committee of the VU University Medical Center, Amsterdam, The Netherlands, approved the study protocol and consent procedures. All patients provided written informed consent.

**Figure 1:**
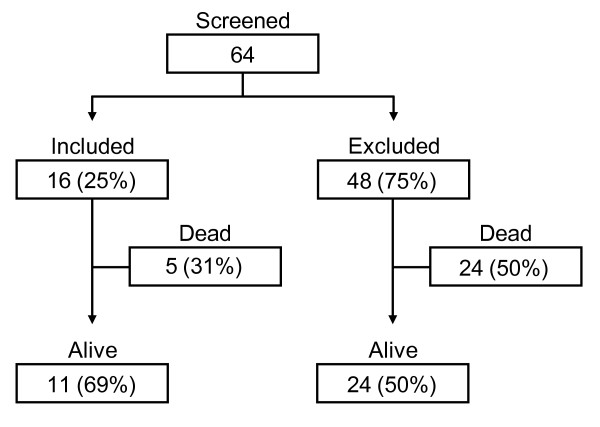
**Flow diagram showing patients screened for enrollment, included/excluded for the peritoneal lavage hypothermia group**. Excluded patients received non-invasively MTH as part of the standard care for these patients. Mortality and survival rates at ICU discharge are incorporated in the flow diagram.

We excluded patients less than 18 years of age, with a core temperature of less than 34°C upon presentation after ROSC, a history of abdominal surgery, peritonitis or currently undergoing peritoneal dialysis, active uncontrolled bleeding or coagulopathy, a known history of receiving thrombolytic medication in the previous six weeks (treatment with other anticoagulant medications, such as those required for percutaneous coronary intervention (PCI) or ST-segment Elevation Myocardial Infarction (STEMI) treatment, is not an exclusion), known significant concomitant illness with a life expectancy of less than one year, known enrollment in another study, or pregnant patients. Figure 1 shows patients screened for enrollment included or excluded for the PL hypothermia group in a flow diagram. Excluded patients received non-invasive MTH as part of the standard care for these patients.

Sedation was maintained using intravenous administration of propofol and fentanyl, and shivering was treated with fentanyl boluses when necessary, according to standard institutional procedure for post-resuscitative therapeutic hypothermia. An EndoTIP peritoneal access port (Karl Storz, Tuttlingen, Germany), commercially available for routine laparoscopic surgery access, was used for blunt (non-cutting) lateral dissection of abdominal wall tissue. The EndoTIP was used in combination with the Peritoneal Entry Indicator ((PEI), Karl Storz, Tuttlingen, Germany), a proprietary fluid reservoir that attaches to the proximal end of the EndoTIP. Entry into the peritoneal cavity was detected by observing the rapid drop of fluid from the PEI into the peritoneal cavity. Once entry was detected, the PEI was removed and a disposable peritoneal catheter was placed through the lumen of the access port. The access port was removed and the catheter was connected to the Velomedix^® ^system. The disposable peritoneal lavage catheter is a multiple lumen device made from a dual lumen extrusion for fluid transfer with a floating third lumen for cavity pressure measurement. During hypothermic treatment, lactated Ringer's irrigation solution (Baxter, Deerfield, Illinois, USA) was continuously infused and extracted through independent lumens in the catheter. An esophageal temperature probe was inserted for continuous temperature monitoring and connected to the system to monitor the subject's temperature during the whole treatment. When a target temperature of 32.5°C was reached, the induction phase was automatically followed by maintenance of hypothermia for 24 hours. Once the maintenance hypothermic treatment time had elapsed, the subject was automatically, gradually warmed to normothermic temperature at a predetermined rate of 0.5°C/h. After the subject's body temperature had returned to 36°C the peritoneal cavity was drained automatically by the system, the catheter was removed and the abdominal entry incision was sutured by the physician.

### Patients in the control group

Prospectively collected data from historical post-CA patients with ROSC admitted to the ICU between July 2008 and August 2010 and eligible for MTH served as controls. The control group consisted of 99 patients who were treated with our standard method of MTH using cooled saline intravenous infusion and cooled blankets (Cincinnati Sub-Zero Medical, Cincinnati, OH, USA) at the VU University Medical Center, Amsterdam, The Netherlands. In the control group, MTH was part of standard care; therefore, informed consent for this group was waived. After admission to the ICU, the patients were covered with cooling blankets and cooling was started. An esophageal temperature probe was inserted for continuous temperature monitoring. The induction phase, carried out until a target temperature of 32.5°C was reached, was followed by the maintenance phase of 24 hours at a temperature of 32.5°C, after which controlled re-warming to 36°C was started.

### Data collection

Prior to and during MTH treatment, details of the CA, initial vital signs (including core temperature), baseline ECG, initial lab work and temperatures were recorded in our patient data monitoring system for critical care (Metavision, iMDsoft^®^, Tel Aviv, Israel). Core body temperature was recorded continuously during induction, maintenance and the re-warming period of the MTH protocol. Maximum temperature variability during the maintenance period was calculated as the difference between the maximum and minimal temperature during 24-hour maintenance. The mean coefficient of variation (VC) for temperature during the maintenance phase was calculated. The Glasgow Coma Scale (GCS) for each patient still alive at the time of hospital discharge was determined. Data on adverse effects, including shivering, mortality, pneumonia, sepsis, significant arrhythmia, renal failure, peritonitis and significant bleeding, within seven days of the procedure were collected through retrospective chart view.

### Statistical analysis

We report continuous variables, which were not generally normally distributed, as medians with 25% to 75% interquartile ranges (IQR). Categorical variables are reported as counts and percentages. To test for significant differences between the two groups, we used the Mann-Whitney *U *test for continuous variables and the chi-squared test for categorical variables. A two-sided *P *≤0.05 was considered to indicate statistical significance.

## Results

A total of 16 comatose patients successfully resuscitated from CA received MTH via PL. The PL group was compared to a control group consisting of 99 post-CA patients treated according to our standard MTH protocol. Table 1 shows the characteristics and outcome of the patients enrolled, while Table 2 shows the cooling time and speed, and temperature data. All patient characteristics and the outcome parameters, length of stay at ICU, days on mechanical ventilation, survival at ICU and neurological outcome at discharge were comparable between the PL and control group (Table 1).

**Table 1:** Patient characteristics and outcome

	Control group (*n *= 99)	PL group (*n *= 16)	*P*-value
Gender, m/f	76/23 (77/23)	14/2 (87/13)	0.65
Age (years)	65 (57 to 73)	62 (57 to 69)	0.47
BMI (kg/m^2^)	25.4 (23.9 to 28.4)	26.6 (24.8 to 30.5)	0.19
OHCA	76 (77)	14 (88)	0.74
Witnessed arrest	63 (64)	11 (69)	0.86
Presenting rhythm VF/VT	43 (43)	10 (77)	0.49
CAG	36 (36)	10 (77)	0.24
PCI	21 (21)	6 (38)	0.37
Length of stay at ICU (h)	119 (75 to 231)	119 (77 to 164)	0.42
Mechanical ventilation (days)	4.0 (3.0 to 7.5)	5.0 (4.0 to 9.5)	0.38
Survival at ICU	46 (46)	11 (69)	0.38
Neuro-outcome at discharge (GCS)	14.0 (10.0 to 15.0)	15.0 (13.5 to 15.0)	0.13

Continuous variables are median (interquartile range) and categorical variables are absolute counts in numbers (percentages). BMI, body mass index; CAG, Coronary angiogram; GCS, Glasgow Coma Scale; OHCA, out of hospital cardiac arrest; PCI, Percutaneous coronary intervention; PL, peritoneal lavage; VF, ventricular fibrillation; VT, ventricular tachycardia

**Table 2:** Mild therapeutic hypothermia timing and temperature data

	Control group	PL group	*P*-value
First temperature measured (°C)	35.0 (34.0 to 35.6)	35.0 (34.9 to 35.7)	0.14
***Induction: ***			
Start time (min)	74 (52 to 130)	169 (137 to 187)*	0.0001
Time to target temperature of 32.5°C (min)	150 (112 to 240)	30 (19 to 60)*	<0.0001
Cooling rate (°C/h)	0.9 (0.5 to 1.3)	4.1 (2.2 to 8.2)*	0.01
***Maintenance:***			
Mean temperature (°C)	32.4 (32.1 to 32.8)	32.4 (32.4 to 32.5)	0.28
Lowest temperature (°C)	31.2 (31.0 to 31.7)	32.2 (32.2 to 32.3)*	<0.0001
Temperature <31°C, n (%)	23 (23)	0 (0)	0.066
Temperature variability (°C)	0.45 (0.38 to 0.70)	2.20 (1.70 to 3.05)*	<0.0001
***Re-warming:***			
Duration to reach 36.5°C (h)	15.7 (11.0 to 23.0)	12.8 (10.0 to 14.8)*	0.005

Continuous variables are median (interquartile range) and categorical variables are counts (percentages). PL, peritoneal lavage. *P ≤0.05 Lavage versus Control group.

**Figure 2:**
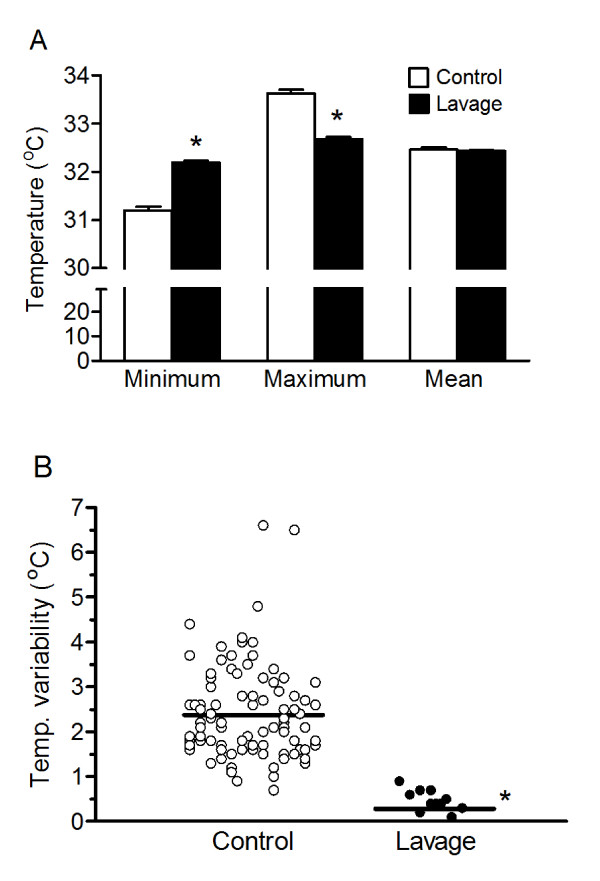
**Temperature control during 24-hour maintenance phase of therapeutic hypothermia**. (**A**) Maximum, minimum and mean temperature during the 24 hour maintenance phase of PL and control groups. Data are mean ± SD. White bars are controls and black bars are PL (Lavage) groups. *P < 0.05 PL versus control group. (**B**) Temperature variability, calculated as difference between maximum and minimum temperature per patient, during the 24-hour maintenance phase in control and PL patients. *P < 0.05 PL group versus control group.

Initial body temperatures were similar between both groups (Table 2). Two out of 16 PL patients were excluded from analysis of temperature parameters because of malfunction of the equipment during the beginning of the maintenance phase. Cooling was initiated after admittance to the ICU. Patients in the PL group reached core target temperatures of 32.5°C significantly faster compared to controls; 30 (IQR: 19 to 60) versus 150 (IQR: 112 to 240) minutes, respectively. A median cooling rate of 4.1°C/h (IQR: 2.2 to 8.2) in the PL group was significantly faster compared to 0.9°C/h (IQR: 0.5 to 1.3) in control patients. In the PL group, cooling started within 169 minutes (IQR: 137 to 187) after arrival at the hospital, which is significantly longer compared to the control group. During the 24-hour maintenance phase mean core temperatures were not different between the groups (Table 2 and Figure 2). However, the largest difference between the minimum and maximum temperature during the 24-hour maintenance phase was significantly less in the PL group (0.45°C with IQR: 0.38 to 0.70) compared to the control group (2.20°C with IQR: 1.70 to 3.05, Table 2 and Figure 2). Also, the coefficient of variation (VC) for temperature during the maintenance phase was lower in the PL group (VC: 0.5%) compared to the control group (VC: 1.5%). None of the patients in the PL group showed an overshoot of hypothermia below 31°C. However, 23% of the control patients displayed core temperatures below 31°C during maintenance, indicating a more steady maintenance phase in the PL group compared to the controls. The re-warming phase in control patients was significantly longer compared to PL patients (15.7 versus 12.8 hours, respectively).

In the PL group, no adverse events or complications related to insertion of the catheter were observed. Device-related skin complications, shivering during MTH, bleeding, peritonitis, pneumonia, sepsis or the need for hemodialysis in the period after MTH was not different between the control and PL group (Table 3). No significant difference was observed in serum creatinine levels between the control and PL groups during the induction, maintenance or re-warming phases of MTH (Table 4). Hematology parameters (Table 4) showed that leukocyte, thrombocyte, PT and aPTT serum levels were not different between the control and PL groups throughout the MTH treatment. In both control and PL groups, leukocyte levels were lower during the maintenance phase and both leukocyte and thrombocyte levels were lower during the re-warming phase compared to the induction phase. Hemoglobin and hematocrit levels were higher in the PL group compared to the control group during the induction phase, unchanged during maintenance and decreased only during re-warming in the PL group. Also, aPTT decreased in the PL group during the re-warming compared to the induction phase. Gastro-intestinal problems or massive inflammatory reaction were not observed in any of the PL patients. However, note that the number of enrolled patients is probably too low to detect complications related to prolonged cooling of the gut.

**Table 3:** Complication rates

Clinical condition	Control group (*n *= 99)	PL group (*n *= 16)
Device related skin complications	3 (3%)	0 (0%)
Shivering (one or more episodes)	2 (2%)	1 (6%)
Bleeding requiring transfusion	4 (4%)	1 (6%)
Peritonitis	0 (0%)	0 (0%)
Pneumonia	4 (4%)	1 (6%)
Sepsis	7 (7%)	1 (6%)
Acute renal failure requiring hemodialysis	14 (14%)	3 (19%)

All values are No. of patients and (%).

**Table 4:** Laboratory values

	Induction phase		Maintenance phase		Re-warming phase	
			
	Control group	PL group	Control group	PL group	Control group	PL group
***Hematology***						
Leucocytes (10^9^/l)	15 (12 to 19)	14 (11 to 16)	12 (9 to 15)†	10 (9 to 13)†	10 (8 to 13)†	9 (8 to 11)†
Thrombocytes (10^9^/l)	207 (168 to 249)	259 (217 to 288)	192 (155 to 227)	197 (183 to 248)	149 (116 to 205)†	167 (152 to 196)†
Hemoglobin (mmol/l)	7.5 (6.5 to 8.6)	8.7 (8.3 to 9.2)*	7.4 (6.5 to 8.5)	8.2 (7.5 to 8.8)	7.1 (6.3 to 7.8)	7.7 (6.8 to 8.3)†
Hematocrit	0.35 (0.31 to 0.42)	0.40 (0.39 to 0.44)*	0.36 (0.31 to 0.41)	0.37 (0.35 to 0.40)	0.35 (0.31 to 0.38)	0.34 (0.33 to 0.38)†
PT (sec)	1.32 (1.19 to 1.57)	1.23 (1.20 to 1.51)	1.30 (1.20 to 1.52)	1.16 (1.15 to 1.28)	1.34 (1.22 to 1.54)	1.25 (1.21 to 1.60)
aPTT (sec)	74 (43 to 164)	107 (79 to 240)	83 (50 to 111)	87 (50 to 109)	68 (55 to 86)	76 (69 to 89)†
***Renal function***						
Serum creatinine (μmol/l)	96 (78 to 125)	96 (89 to 99)	89 (62 to 134)	92 (79 to 105)	100 (65 to 155)	113 (78 to 151)

Continuous values are presented as median (interquartile range). Parameters are presented as average for each therapeutic hypothermia phase. * *P *< 0.05 versus corresponding control group, † *P *< 0.05 versus corresponding group in induction phase.

## Discussion

The present study investigated for the first time the safety and feasibility of rapid induction of MTH via peritoneal lavage using the Velomedix^® ^automated PL system in CA patients after successful resuscitation. We demonstrate that PL is an ultra-rapid and safe cooling technique for reaching and maintaining post-CA patients at a target temperature of 32.5°C for a period of 24 hours. The main findings were as follows: (i) PL resulted in a significantly faster time to reach core target temperatures of 32.5°C; (ii) patients in the PL group showed less variation in temperature during the 24-hour maintenance phase; and (iii) the PL group had a significantly shorter and more controlled re-warming phase compared to control patients who were cooled with saline intravenous infusion and cooled blankets. Furthermore, in our cohort no serious complications related to the therapy and procedure were observed.

Over the past decade several randomized- and historical-controlled trials have shown beneficial effects of MTH on neurological outcome and survival in patients after CA [2,3,16-19]. However, these trials have not yet shown clear evidence for the optimal timing of initiating MTH, rate of cooling and time to reach the target temperature after CA. So far, the benefits of early or rapid cooling have only been demonstrated in animal models testing the effects of brief periods of therapeutic hypothermia. These studies showed that even a minor temperature decrease before, during or immediately after the time of cardiac arrest was associated with benefits in neurological outcome [10,20-22]. Although no prospective randomized clinical trials have assessed the effect of time to reach target temperature on neurological outcome, there are signs that support a benefit to rapid cooling. Wolff *et al. *[23] studied rapidly cooled patients, using an endovascular device, and showed that the time to target temperature was an independent predictor of good outcome. Although underpowered, recent clinical trials of rapid cooling devices have shown trends towards survival benefit [24] and improvement in patient outcomes compared to historical controls [13]. Kory *et al. *[25] published a fast cooling method, using a combination of core and surface cooling modalities without the use of a commercial device, which resulted in a cooling rate of 2.6°C/h. Moreover, cooling via gastric lavage is a technique used to achieve rapid cooling of hyperthermic patients [26]. Unfortunately, none of these cooling methods seem to be superior in generating an ultra-rapid cooling speed. Merchant *et al. *[27] have expressed concern that temperature overshoot below 32°C may be associated with adverse hemodynamic and arrhythmic effects. In our study we showed that in contrast to 24% of the control patients, none of the patients in the PL group reached temperatures below 31°C.

Recently, Haugk *et al. *[14] reported a retrospective analysis of 13 years of therapeutic hypothermia patients, suggesting a surprising association between faster cooling rates and less favourable neurological outcomes. The authors suggested that their findings might well imply that greater neurological injury is predicted by more rapid achievement of cooling during therapeutic hypothermia, rather than rapid cooling itself being inherently harmful. In patients with poor neurological outcomes, the initial temperatures were significantly lower before therapeutic hypothermia was started, suggesting that these patients already show some degree of impaired thermoregulation. Alternatively, it appears that sicker patients lack homeostatic mechanisms to maintain normothermia and, therefore, cool faster. In our study, temperatures at the start of cooling were not different between the two groups. The five-times faster cooling rate in the PL group (4.7°C/h) compared to the control group (0.9°C/h) can, therefore, not be attributed to impaired thermoregulation.

### Limitations

This study, reporting on the first experience with a new technique at a single institution, is a controlled trial, but with the use of historical controls. Selection bias is present in this study due to the exclusion of patients with a history of abdominal surgery and the inclusion of a relatively small number of patients. Therefore, our results of ultra-fast cooling via PL may not be translated directly to other hospitals.

Also, the time interval from hospitalization to target temperature is mainly dependent upon team organization and the technique used to induce MTH. On average, it took 168 minutes after hospitalization to start with the cooling therapy and after cooling was started only 30 minutes to achieve the target temperature in the PL group. The time to initiation of cooling was longer in the PL group compared to the control group, but target temperature was reached much faster than in the controls. This can be explained by the period needed to obtain informed consent from a legal representative, which is necessary for the PL patients. Secondly, insertion of the PL catheter by a physician is at present somewhat longer then applying cooled intravenous infusion and blankets on the patient as used in the control group. Apparently, there is still a great potential to further optimize the period between hospitalization and the start of cooling and thereby speed up the whole PL procedure. One might initiate PL in the emergency room, requiring, however, reorganization and in depth-training of the cardiopulmonary resuscitation team. In this study, we decided to test this new technique in a controlled setting in the ICU. When this technique is applied as a routine technique in the future, the timing delay to start cooling will not be an issue. Finally, some bias could have occurred in the patient population of the PL group due to strict exclusion criteria. However, patient characteristics were equal between control and PL patients.

### Applications

So far, only animal studies have shown benefits of faster cooling rates; translation to clinical care has not yet proved these benefits. Nowadays, one of the greatest challenges is the introduction of rapid cooling methods achieved in laboratory animals to human subjects, with the hopes of observing similar therapeutic benefits. The results of the present clinical study showed that PL resulted in an extremely fast cooling rate and steady maintenance phase during MTH therapy. These observations provide a unique opportunity to investigate the effects of ultra-rapid cooling on neurological outcome and survival in prospective controlled trials in various patient groups; such as its use in emergent situations, where there is a possible benefit of inducing MTH prior to adjunct therapy, or in the case of percutaneous coronary revascularization to reduce infarct size following acute myocardial infarction. Furthermore, another group of patients who can benefit from ultra-rapid cooling are patients with acute neurological injuries, such as stroke.

## Conclusions

This study shows the first experience with a novel technique for induction of MTH. In a short period of time a substantial number of patients were included, which demonstrates the feasibility of this novel technique. The results demonstrate evidence for an extremely rapid, safe and feasible way of applying MTH via peritoneal lavage in a controlled ICU tting compared to historical controls. Whether this also holds in the emergency room or in a cardiology environment, during the acute post-cardiopulmonary resuscitation phase, remains to be determined.

## Key messages

• Peritoneal lavage is an ultra-rapid technique to achieve mild therapeutic hypothermia in patients with ROSC after CA.

• Peritoneal lavage shows a very stable temperature control during the 24-hour maintenance phase of MTH.

## Abbreviations

CA: cardiac arrest; ECG: electrocardiography; GCS: Glasgow Coma Scale; ICU: intensive care unit; IQR: interquartile range; MTH: mild therapeutic hypothermia; PCI: percutaneous coronary intervention; PEI: peritoneal entry indicator; PL: peritoneal lavage; ROSC: return of spontaneous circulation; STEMI: ST-segment elevation myocardial infarction; VC: coefficient of variation

## Competing interests

The authors declare that they do not have any competing interests. The VU University Medical Center, Amsterdam received a fee per included patient from Velomedix (manufacturer of the device and sponsor of the CAMARO clinical study). Velomedix did not finance this manuscript or data processing nor pay the article-processing charge to VU University Medical Center or any of the authors.

## Authors' contributions

MW collected and interpreted the data, performed the statistical analysis and drafted the manuscript. HB, SB, YA, RD, KP, AG and AB participated in the study design. HB and AB performed the abdominal catheterizations and the experimental procedures. RD collected the data. All authors read and approved the final manuscript.
